# Evaluation of pest control contributions to the production of three major grain crops in China

**DOI:** 10.1007/s44297-025-00060-5

**Published:** 2025-12-08

**Authors:** Hui Liu, Xiaoming Zhu, Fuyan Zhuo, Yue Li, Fuxiang Wang, Ping Li, Zhaoyue Ge, Yongwang Guo, Wancai Liu

**Affiliations:** National Agricultural Technology Extension Service Center/National Agricultural Technology Integration and Innovation Center, Beijing, China

**Keywords:** Grain crops, Pest prevention and control, Contribution of plant protection

## Abstract

Wheat, rice and maize are three major grain crops in China. In 2024, under the organization of National Agricultural Technology Extension Service Center, the contribution rates of controlling pests (diseases, insect pests and weeds) on these crops to their production were evaluated in 15 provinces of the country. In each province, five control treatments for the pests were set up at different locations, including strict control, unified control, traditional farmer control, no control of diseases or insect pests, and no control of diseases, insect pests or weeds. In the plots of each treatment, yields and losses were measured and compared between treatments. The results revealed that the average contribution rate of pest control in China in 2024 was 30.56% for the three crops, reaching 27.99%, 35.62% and 28.63% for wheat, rice and maize, respectively, and the recovered yield losses were 39.21, 73.92, and 84.43 million tons, respectively, totaling nearly 198 million tons. The contribution rate was higher when the pests were controlled under strict guidelines and when the control was complemented in a unified form than when the pests were controlled via methods freely adopted by farmers. Moreover, the damage caused by weeds tended to increase, which was most serious in paddy fields but much less severe in wheat fields.

Article 24 of Chapter 3 of the *Grain Security Guarantee Law of the People's Republic of China* stipulates that "It is important to strengthen the prevention and control of diseases and insect pests of grain crops and plant quarantine", and “The state encourages and supports the development of green prevention and control of crop diseases and insect pests, and their unified control". This is the first time that China has made it clear, at the legal level, that strengthening pest control is an important measure to ensure the safety of national food production. To achieve this goal to reduce yield losses, in recent years, the Ministry of Agriculture and Rural Areas has taken a series of measures by organizing agricultural and rural departments and plant protection agencies throughout the country. The measures target serious diseases and insect pests of grain and oil crops, such as wheat, rice and maize. As an outstanding result, outbreaks of some major pests have been extensively suppressed, and their damage and yield losses have been reduced to a large degree, thereby promoting the stable and high-yield production of grain crops.

To evaluate the role of disease and insect pest prevention and control in ensuring food security, the National Agricultural Technology Extension Service Center (NATESC) has issued *measures for evaluating the contribution rate of plant protection to the prevention and control of crop diseases and pests*. Moreover, under the leadership of NATESC, plant protection departments and agencies began testing the effectiveness of these evaluation measures nationwide in 2022. The overall contribution rates of pest prevention and control (excluding weeds and rodents) in 2022 to grain production were estimated to be 20.31%, accounting for 24.20%, 19.46% and 18.84% for wheat, rice and maize, respectively [1]. In 2023, a blank control, i.e., without controlling against pests, was added to obtain a more reasonable result, and the contribution rate increased to 30.59%, reaching 27.58%, 40.58% and 25.66% for wheat, rice and maize, respectively [2]. As the acreage of crops suffering pest damage, levels of damage, and control measures undertaken may vary among years, evaluations have to be performed for more years. For this reason, in 2024, NATESC extended the evaluation to a larger range, covering 149 locations in 144 counties (cities and districts) of 15 provinces. In this year, the contribution rates for the three major grains were 30.56%, 27.99%, 35.62% and 28.63% for wheat, rice and maize, respectively, and their yield losses were reduced by 39.21, 73.92 and 84.43 million tons, respectively, for a total of nearly 198 million tons.

## Methods

### Geographic division of the evaluation events

The provinces and counties regionally representative of the targeted crop(s) were selected for evaluation, as listed in Table 1. When these provinces are selected, their ability to protect plants and manage pests should be considered for evaluation as solid as possible. Three grain crops were included in the evaluation, i.e., wheat, rice and maize, and the selected provinces included all of the major provinces of the country cultivating them. For each crop, the selected locations covered most of its dominant growing regions in the country. In each of the selected provinces, at least four counties (or districts) implemented the evaluation, which represented different cultivation systems or modes in the province (Table 1). Each of these counties was taken as an independent experimental unit of evaluation.

**Table 1:** Locations selected for the evaluation of the contribution rates of plant protection in China in 2024

Crops	Provinces	Counties (districts)
Wheat	Hebei	Zhaoxian, Luancheng, Wuyi, Boye;
	Anhui	Fengyang, Jieshou, Lingbi, Wuhe, Changfeng;
	Shandong	Bincheng, Zhangqiu, Pingyuan, Yanggu and Heze
	Henan	Dengzhou, Tanghe, Zhenping, Gushi, Yancheng, Changge, Huaiyang, Dancheng, Nanle, Yanjin;
	Hubei	Anlu, Suixian, Tianmen, Xiantao, Zhongxiang, Songzi;
Rice	Heilongjiang	Fangzheng, Tailai, Jidong, Hulin, Raohe, Suiling;
	Jiangsu	Dafeng, Jingjiang, Tongzhou, Suining, Taicang and Yixing;
	Zhejiang	Qujiang, Anji, Jiangshan, Longyou, Tongxiang and Changxing;
	Henan	Gushi, Guangshan, Huangchuan, yuanyang and Changyuan;
	Jiangxi	Taihe, Zhangshu, Anyuan, Ruichang, Fuzhou Linchuan, Wuyuan;
	Hubei	Qichun, Chibi, Huangmei, Yangxin, Jingshan, Nanzhang, Dangyang, Qianjiang, Yingcheng, Jianli;
	Hunan	Ningxiang, Hengdong, Hengnan, Chaling, Xiangxiang, Shaodong, Linxiang, Huarong, Lixian, Zhangjiajie Yongding District, Nanxian County, Guiyang, Dongan, Tongdao, Lianyuan, Xinhua and Yongshun;
	Guangdong	Conghua, Huazhou, Kaiping, Renhua, Lufeng;
	Chongqing	Dianjiang, Nanchuan, Yongchuan, Hechuan, Jiangjin, Zhongxian, Xiushan;
	Szechwan	Qionglai, Zhongjiang, Cangxi and Luxian;
Corn	Hebei	Quzhou, Pingxiang, Jingxian, Zhengding
	Jilin	Siping, Yushu, Dunhua, Shulan, Fusong, Dongliao and Taonan
	Heilongjiang	Shuangcheng, Meilisi, Muling, Mishan, Zhaozhou and Anda
	Anhui	Guzhen, Mengcheng, Qiaocheng, Xiaoxian, Lixin
	Henan	Huaiyang, Boai, Huojia, Jiyuan, Changge, Yanjin, Xiayi, Pingyu, Shangshui, Xingyang
	Hubei	Zhushan, Fangxian, Badong, Lichuan, Xiangzhou, Zaoyang, Zhongxiang, Xiantao and Zhijiang
	Shaanxi	Gaoling, Qishan, Linyou, Fuping, Yongshou, Yuyang

### Design of the evaluations

In each of the selected counties, five different control levels (treatments) were adopted to create a gradient of disease and insect pest occurrence. a) The pests were controlled under the strict supervision of a plant protection technician (strict control); b) they were controlled with unified rules (unified control); c) they were controlled by methods freely adopted by farmers (traditional control); d) diseases, insect pests and weeds were not controlled; and e) diseases and insect pests were not controlled while weeds were controlled. Treatments *d* and *e* served as controls, 1/15 ha. Each, with one replicate, is expected to reach the most severe damage level (i.e., highest yield losses). The other three treatments were 134–200 m^2^ each, with three replicates. In treatment *a*, the damage level was hypothesized to be the lowest, and in *b* and *c*, there was medium damage.

### Contribution rates of plant protection at different control levels

During the crop harvest period, the yields in the plots of different treatments were measured. The rates of yield losses caused by insect pests and diseases and the rates of losses recovered from the control were subsequently calculated via formulas (1) to (3).

1 $$\text{Maximum loss rate }\left({\%}\right)=(\text{yield per unit area with strict control}-\text{yield per unit area with no control})/\text{yield per unit area with strict control}\times 100$$

2 $$\text{Actual loss rate }\left({\%}\right)=(\text{yield per unit area with strict control}-\text{yield per unit area with treatments b},\text{ c},\text{ d or e})/\text{yield per unit area with strict control}\times 100$$

3 $$\text{Recovery loss rate }\left({\%}\right)=(\text{yield per unit area with treatments b},\text{ c},\text{ d or e}-\text{yield per unit area with no control})/\text{yield per unit area with strict control}\times 100$$

Next, the contribution rate of plant protection (PP) at different control levels was calculated via formula (4). This can also be represented by the recovery loss rate (formula 3).

4 $$\text{PP contribution rate }\left({\%}\right)=\text{Maximum loss rate}-\text{Actual loss rate}$$

### Contribution rates of plant protection at different administrative levels

For each selected county, we recorded the geographic information for each deployed control treatment (*a* ~ *e*), levels of pest occurrence, and proportions of the acreage at each of these occurrence levels in the total cropping acreage (termed the “acreage proportion” hereafter). The pest occurrence levels for wheat, rice and maize were assessed, and then, the proportions of representative areas receiving strict control, unified control, and traditional farmer control were calculated separately.

The weighted average method was used to calculate county-level PP contribution rates, which were weighted by the proportion of cultivation area (CA) at different pest occurrence levels.

5 $$\text{PP contribution rates at the county level }\left({\%}\right)=\sum [\text{yield per unit area with treatments b},\text{ c},\text{ d or e}-\text{yield per unit area with no control})/\text{yield per unit area with strict control}\times \text{proportion of CA at the pest occurrence level}]$$

Similarly, the PP contribution rates at the city level were calculated via the methods described above. They could also be calculated by weighting the PP contribution rates of each involved county. The PP contribution rates at the province level were calculated by averaging the contribution rates of 5–7 counties, weighted by the ratio of CA in each of these counties to their pooled CA. The methods were similar for the calculation of the station-level PP contribution, weighted by the ratio of CA in each province to the pooled CA.

6 $$\text{PP contribution rate at the province level }\left({\%}\right)=\sum (\text{PP county}-\text{level contribution rate}\times \text{ratio of CA of the county to the pooled CA})\times 100$$

7 $$\text{National PP contribution rate }\left({\%}\right)=\sum (\text{PP province}-\text{level contribution rate}\times \text{ratio of CA in the province to the pooled CA})\times 100$$

### Assessment of the overall contribution rate of plant protection for grain crops in China

The overall PP contribution in the country to grain crop production was calculated according to the rates obtained from (8) for each of the three crops (wheat, rice and corn) and the ratio of their corresponding CA to the pooled CA.

8 $$\text{Overall national PP contribution }\left({\%}\right)=\sum (\text{National PP contribution rate of a crop}\times \text{Ratio of the national CA of the crop})$$

### Yield loss rate due to weed damage

The damage caused by weeds was calculated via the following formula:

9 $$\text{Yield loss from weed damage }\left({\%}\right)=(\text{loss rate in treatments not controlling disease},\text{ insect pests and weeds})-(\text{loss rate in treatments not controlling disease and insect pests})$$

## Results

### Contribution rates of disease control and insect pest control to wheat production

According to the data from the five major wheat-producing provinces, i.e., Hebei, Anhui, Shandong, Henan and Hubei, the national contribution rate of plant protection to wheat production reached 27.99%. Specifically, the rates attained under strict controls and unified controls are 11.73% and 5.36% higher than those from traditional farmer controls, respectively (Table 2).

**Table 2:** Contribution rates of plant protection to the production of wheat in China in 2024

Provinces	Strict control areas		Unified control areas		Farmers’ traditional control areas		Provincial contribution rate (%)	National contribution rate (%)
	Recovery loss rate (%)	Area ratio (%)	Recovery loss rate (%)	Area ratio (%)	Recovery loss rate (%)	Area ratio (%)		
Hebei	32.98	14.13	27.93	72.37	26.63	13.5	28.47	27.99
Anhui	37.25	10.00	28.52	71.22	25.50	18.78	28.82	
Shandong	32.37	7.27	29.16	56.87	23.91	35.86	27.51	
Henan	33.58	11.58	28.88	58.48	13.57	29.89	27.70	
Hubei	38.91	3.25	28.73	46.53	26.82	50.22	28.10	
Average	35.02	−	28.64	−	23.29	−	28.12	

### Contribution rates of disease control and insect pest control to rice production

Among the ten provinces, Heilongjiang, Jiangsu, Zhejiang, Henan, Jiangxi, Hubei, Hunan, Guangdong, Chongqing, and Sichuan, the contribution rate to rice production ranged from 20.13% to 46.33%, and the rate at the national level reached 35.62%. The rates in the strict control and unified control treatment groups were higher by 6.69% and 5.43%, respectively, than those in the traditional control group (Table 3).

**Table 3:** Contribution rates of plant protection to the production of rice in China in 2024

Provinces	Strict control areas		Unified control areas		Farmers’ traditional control areas		Provincial contribution rates (%)	National contribution rates (%)
	Recovery loss rate (%)	Area ratio (%)	Recovery loss rate (%)	Area ratio (%)	Recovery loss rate (%)	Area ratio (%)		
Helongjiang	―	―	49.72	41.06	46.04	48.62	45.77	35.62
Jiangsu	46.45	12.37	42.32	64.81	38.11	21.91	41.71	
Zhejiang	44.96	2.06	39.28	60.5	38.49	36	38.55	
Jiangxi	46.61	1.66	49.41	67.05	45.46	31.29	46.33	
Henan	32.95	5.16	30.76	50.24	18.39	44.6	25.36	
Hubei	38.57	3.55	34.34	46.33	28.11	50.14	31.37	
Hunan	41.96	3.35	37.88	54.43	31.14	42.22	35.17	
Guangdong	32.6	6.96	29.04	53.21	24	39.3	27.17	
Chongqing	32.59	8	24.92	40	19.69	52	22.81	
Sichwan	27.71	8.5	26.7	57.9	20.87	27.3	20.13	
Average	38.98		37.72		32.29		34.62	

### Contribution rates of disease control and insect pest control to corn production

The contribution rate to corn production in the seven provinces selected, i.e., Hebei, Jilin, Heilongjiang, Anhui, Henan, Hubei and Shaanxi, was between 20.08% and 35.61%, and at the national level, it was 28.63%. The contribution rates in the strict-control and unified-control treatments were 30.40% and 29.47%, respectively, which were 7.86% and 6.93% higher than those (22.54%) in the traditional control treatments (Table 4).

**Table 4:** Contribution rates of plant protection to the production of corn in China in 2024

Provinces	Strict control areas		Unified control areas		Farmers’ traditional control areas		Provincial contribution rates (%)	National contribution rates (%)
	Recovery loss rate (%)	Area ratio (%)	Recovery loss rate (%)	Area ratio (%)	Recovery loss rate (%)	Area ratio (%)		
Hebei	31.11	13.88	29.12	63.75	24.21	22.37	28.30	28.63
Jilin	28.32	12.41	25.13	46.86	23.75	37.19	24.93	
Helongjiang	―	―	48.83	12.93	37.00	57.39	35.61	
Anhui	31.70	16.50	26.1	52.3	17.3	30.3	24.19	
Henan	35.52	11.49	28.69	59.10	18.38	29.41	26.44	
Hubei	31.98	2.11	26.9	46.1	22.43	51.79	24.69	
Shaanxi	23.76	24.25	21.51	48.01	14.74	24.15	20.08	
Average	30.40		29.47		22.54		26.32	

### Overall contribution rates of disease control and insect pest control to grain crop production in China

In 2024, the cultivation areas of wheat, rice and corn accounted for 24.41%, 29.91% and 45.68%, respectively, of the pooled cultivation area of these crops. On the basis of these data and the contribution rates described above for each crop (i.e., 27.99%, 35.62% and 28.63%, respectively), the overall contribution rate of plant protection for grain crop production was assessed to be 30.56%, as calculated according to formula (8). By strengthening the control of diseases and insect pests, the losses of wheat, rice and maize were reduced by 39.2137, 73.9240 and 84.4347 million tons, respectively, totaling nearly 198 million tons (Table 5).

**Table 5:** Contribution rates of plant protection to the production of grain crops in China in 2024

Crops	Contribution rate for the crop (%)	Yield (m. tons)	Recovered production (m. tons)	Cultivation area (m. ha.)	Proportion of cultivation area (%)	Overall contribution rate (%)
Wheat	27.99	140.099	39.2137	354.40	24.41	30.56
Rice	35.62	207.535	73.9240	434.23	29.91	
Corn	28.63	294.917	84.4347	663.28	45.68	
Average/Total	30.75	642.551	197.5724	1451.91	100	

### Assessment of weed damage and estimated yield loss for the three major grain crops

By setting up two treatments, i.e., no control against diseases, insect pests or weeds and no control against diseases or insect pests while the weeds were controlled, the loss resulting from weed damage was estimated for each crop. The losses in wheat, rice and corn ranged from 5.9%−13.5%, 4.9%−33.7%, and 6.6%−32.6%, with averages of 8.11%, 14.34% and 14.74%, respectively. The losses varied tremendously among regions, with the highest losses of wheat, rice and corn occurring in Anhui (13.47%), Heilongjiang (33.65%) and Heilongjiang (32.61%). By calculating the proportion of the loss rate caused by weed damage to the total crop loss rate (caused by diseases, pests, and weeds), the severity of weed harm in different regions can be clearly determined (Table 6).

**Table 6:** Rates of yield losses caused by weeds in 2024 in different regions of China

Crops	Provinces	Weed-caused yield loss rate (%)	Proportion of weeds-caused loss in the total loss (%)	Average loss (%)
Wheat	Hebei	8.54	25.89	8.11
	Anhui	13.47	36.16	
	Shandong	6.07	18.75	
	Henan	5.88	17.51	
	Hubei	6.60	16.96	
Rice	Heilongjiang	33.65	67.68	14.34
	Jiangsu	21.75	46.82	
	Zhejiang	21.91	48.73	
	Jiangxi	15.19	29.34	
	Henan	5.64	17.12	
	Hubei	11.24	29.14	
	Hunan	13.04	31.08	
	Guangdong	4.85	14.88	
	Chongqing	9.42	28.90	
	Szechwan	6.68	27.68	
Corn	Hebei	10.93	35.13	14.74
	Jilin	23.08	81.50	
	Heilongjiang	32.61	66.78	
	Anhui	7.30	23.03	
	Henan	6.57	18.50	
	Hubei	7.97	24.92	

## Discussion

### Effective prevention and control of diseases, insect pests and weeds is critical for reducing yield losses

For each of the three crops, the plant protection contribution rates were higher under strict control and unified control than under traditional farming practices and higher under strict control than under unified control (Tables 2, 3 and 4). This suggests that effective prevention and control of diseases and insect pests is rather effective for reducing yield losses, and this can be realized by strengthening the technical guidance for the controls and further unifying the control methods and procedures. Since different crops are distributed across varying regions and thus may suffer different levels of damage, we used a weighted calculation method in the analysis, which is more reasonable than directly averaging the contribution rates across crops. Moreover, the control measures were implemented starting from the seed treatment stage (prior to sowing) and continued across the whole growing season rather than being limited to only a few growth stages. This approach also enhances the reliability of our evaluation.

### Weed damage has increased in recent years

Weeds, as one of the major pest groups, can cause large yield losses in each of the three major grain crops in China (Table 6). However, their damage level varies among crops, being lower in wheat fields (8.11% on average) and more severe in rice and corn fields (nearly 15% both). Their damage also varies over time and has tended to worsen in recent years. For example, compared with the situation in 2023, the weed-caused yield loss was greater in 2024 for each of the three crops, although such a difference might also partially result from the interyear changes in the locations under evaluation. This is also the primary reason why, starting in 2023, we set up the two treatments described above (*d* and *e*) to evaluate weed loss rates.

Weed damage also exhibits evident regional variance. In both rice and corn fields, weed infestations are largely more severe in the northeastern region, where it is the primary source of damage in local fields. In contrast, some areas, such as the Huang-Huai region, experienced much less damage. Particularly in corn fields, the yield loss caused by weeds was only 7–11%, except for Heilongjiang and Jilin. The light damage in these areas may be attributed to the greater height of corn plants in later seasons, which possibly suppresses the growth of weeds and thus reduces their impacts. In wheat fields, weed damage is similar in Shandong and Henan, but it is greater in Anhui, where a rice‒wheat rotation system is adopted, as observed in both 2023 and 2024. In addition, regions in proximity tend to have similar weed damage levels, which is observed in Jiangsu and Zhejiang, as well as in Jiangxi and Hunan, both of which are double-cropping rice regions.

### Inter-year and regional comparisons of plant protection contribution rates

Beginning in 2022, NATESC began to conduct an evaluation of plant protection contributions across a large geographic range of the country. For wheat, the evaluation was carried out for three consecutive years in Hebei, Anhui, Shandong, and Henan Provinces, with Hubei added in 2024. In each of these provinces, the selected sites were adjusted annually. We found that the contribution rates in 2023 (27.58%) and 2024 (27.99%) were quite similar.

For rice, the provinces and sites selected for evaluation varied greatly between years. Some major grain-producing provinces, such as Heilongjiang, Jiangsu, Jiangxi, Hunan, and Sichuan, were selected each year. In 2023, more locations were used for analysis, which expanded to nine provinces and 60 counties (cities, districts). In 2024, three other provinces, Henan, Hubei, and Guangdong, were added, but Anhui, Guangxi, and Chongqing were excluded. The plant protection contribution rates for 2023 and 2024 reached 40.58% and 35.62%, respectively, and were significantly lower in 2024. This difference may be attributed primarily to adjustments in the provinces included in the analysis. For example, in 2024, Anhui experienced severe damage from rice stem borers and weeds, with a rather high contribution rate of 65.72%; however, we did not use these data because we included data from two provinces, Jiangsu and Zhejiang, which neighbor Anhui. On the other hand, within rice-growing regions, the contribution rate changes little between 2023 and 2024, as seen in Heilongjiang, Jiangsu, and Hunan, as well as in Guangdong and Guangxi, both of which are located in the southern rice-growing region. We also found evident interyear differences in some regions, such as Jiangxi and Hunan, where rice is grown in both single- and double-cropping seasons. In such regions, rice plants of various stages coexist, and various cultivation methods (e.g., direct seeding and transplanting) are used, which may lead to tremendous interyear spatiotemporal changes in disease, insect pest and weed occurrence or damage.

### Composition and accuracy of the plant protection contribution rates

The plant protection contribution rates presented in this study reflect not only the losses mitigated by control measures implemented after sowing but also the outcomes of preventive measures taken before sowing. For example, in recent years, seed treatments with chemicals have been applied before sowing in many regions to prevent wheat stripe rust and wheat crown rot [3, 4]. Liu et al. (2024) argued that the high plant protection contribution rate for wheat stripe rust control in China was achieved on the basis of the integrated use of various, but not a few, preventive measures in recent years [5]. Similarly, a part of the contribution rate for rice can also be attributed to the controls implemented during the nursery cultivation of seedlings.

Further studies are needed to obtain more accurate plant protection contribution rates. During the evaluations reported here, the areas of some blank controls were slightly small. Additionally, some control plots were located close to other treatment plots so that some pests in the treatment plots might have spread to the control plots. The impacts of these factors should be reduced in future evaluations.

## Data Availability

All data supporting the findings are available in the paper.
